# Face masks affect emotion categorisation, age estimation, recognition, and gender classification from faces

**DOI:** 10.1186/s41235-022-00438-x

**Published:** 2022-10-08

**Authors:** Hoo Keat Wong, Alejandro J. Estudillo

**Affiliations:** 1 School of Psychology, University of Nottingham Malaysia, Semenyih, Selangor Malaysia; 2 Department of Psychology, Bournemouth University, Bournemouth, UK

**Keywords:** Age estimation, COVID-19, Emotion recognition, Face coverings, Face recognition, Gender classification

## Abstract

Although putting on a mask over our nose and mouth is a simple but powerful way to protect ourselves and others during a pandemic, face masks may interfere with how we perceive and recognize one another, and hence, may have far-reaching impacts on communication and social interactions. To date, it remains relatively unknown the extent to which wearing a face mask that conceals the bottom part of the face affects the extraction of different facial information. To address this question, we compared young adults’ performance between masked and unmasked faces in four different tasks: (1) emotion recognition task, (2) famous face recognition and naming test, (3) age estimation task, and (4) gender classification task. Results revealed that the presence of face mask has a negative impact on famous face recognition and emotion recognition, but to a smaller extent on age estimation and gender classification tasks. More interestingly, we observed a female advantage in the famous face recognition and emotion recognition tasks and a female own-gender bias in gender categorisation and age estimation tasks. Overall, these findings allude to the lack of malleability of the adulthood face recognition and perceptual systems.

## Introduction

Since the unprecedented COVID-19 pandemic outbreak in 2019, wearing face masks has become a prevalent practice for curbing the spread of coronavirus. Some countries made it mandatory to wear masks when in public. While face masks are one of the best defences against the spread of respiratory virus, their growing adoption has posed a challenge not only to the way people interact with each other in social situations (Eikenberry et al., 2020), but also to human face recognition and perception (Carbon, 2020; Carragher & Hancock, 2020; Estudillo et al., 2021; Freud et al., 2020). Following the widespread use of face masks, the fundamental concerns pertain to difficulties in extracting facial identity and making social judgements on one’s characteristics (e.g. age, gender, and emotion) from faces during daily interactions (Fitousi et al., 2021), which may reduce adherence to face mask regulation (Fortin, 2020).

Bruce and Young’s (1986) functional model of face recognition suggests that unfamiliar and familiar face recognition falls along different routes. Recognition of unfamiliar faces involves structural encoding of faces, directed visual processing, as well as facial expression and speech analysis. Structural encoding of faces ensures the formation of a quick and basic description of faces and directed visual processing allows the quick extraction of physical aspects of faces such as age, gender and race. In addition to that, recognition of familiar faces involves the activation of face recognition unit (FRU), followed by the person identity node (PIN), and then name generation. Successful recognition of familiar faces is achieved when there is a match between the products of accurate structural encoding and previously stored representations of familiar faces, held in the FRUs. According to this functional model of face recognition, facial identity and emotional expression are processed in parallel and independently. Here, we investigated whether the four aspects of face processing that tap into different subcomponents of the model—(1) *famous face recognition* via recognition route (i.e. FRU → PIN → name generation); (2) *emotion recognition* via expression analysis; (3) *gender decision* and (4) *age estimation* via directed visual processing—are affected differentially by the presence of face mask.

Face recognition is probably humans’ most basic social skill. Although face recognition ability is used extensively in our daily life, the common difficulty in identifying unfamiliar faces (Bruce et al., 1999; Estudillo, 2012) contrasts with the ease with which most people recognise familiar faces (Young et al., 1985). It is well-established in the memory literature that recognition of familiar and unfamiliar faces differs in a number of ways (for reviews see Johnston & Edmonds, 2009; Young & Burton, 2017) and external features may dominate face-matching decisions when recognising unfamiliar faces compared to familiar faces (Ellis et al., 1979; Wong et al., 2020).

The recent outbreak of Covid-19 pandemic has garnered great research interest on the face mask effect—the occlusion of the lower part of the face hinders the identification of familiar faces (Carragher & Hancock, 2020) and unfamiliar faces (Carragher & Hancock, 2020; Estudillo et al., 2021; Freud et al., 2020; Noyes et al., 2021; Stajduhar et al., 2021). For example, Freud et al. (2020) studied how masks impair people’s facial recognition abilities. They recruited nearly 500 adults to complete the classic Cambridge Face Memory Task (CFMT; Duchaine & Nakayama, 2006) online. Participants viewed *unfamiliar* faces and then tried to recognise them under increasingly difficult conditions. Half of the participants were presented with faces with surgical masks covering their mouths and noses. Expectedly, participants performed substantially worse on the test when faces were masked. More specifically, 13% of their participants struggled to recognise masked faces to the extent as if they have suffered from prosopagnosia. Without masks, only 3.5% scored that low. One possible cause of this impairment is that covering the lower half of the face reduces the availability of holistic information and the processing of the spatial relations among internal features (e.g. the distance between nose and upper lip).

In other online familiar face-matching tests (Carragher & Hancock, 2020), people performed worse when masks are superimposed onto the faces—even when the faces belonged to familiar celebrities; however, compared to Freud et al., the magnitude of face mask effect was much smaller, likely due to a lower reliance of view-dependent facial representation (Estudillo & Bindemann, 2014). That is, familiar face recognition is mediated by more abstract structural codes that define facial identity, allowing generalization across different expressions, viewpoints, and lighting conditions. In contrast, unfamiliar face memory is reliant on view-dependent representation, likely to be dominated by pictorial cues (e.g. hairstyle, freckles).

Although people seem to be able to recognise familiar faces effortlessly despite a diverse range of adverse conditions, putting on a mask over the nose and mouth may subtly interfere with how we recognise faces which are familiar on an individual level (Carragher & Hancock, 2020; Freud et al., 2020; Noyes et al., 2021). Although previous studies have shown that face masks affect performance on familiar and unfamiliar face matching, research on how covering the lower half of the face with protective masks affects performance on famous face recognition is scarce. Noyes et al. (2021) recently reported that face identification and emotion recognition appear relatively robust against occlusion by face mask, which is no worse than the results for faces wearing sunglasses. However, unlike the face matching task that could be solved by using a piecemeal, feature-by-feature matching strategy (Bindemann, 2021), famous face recognition requires participants to access identity information in long-term memory in order to match an input face with a viewpoint-independent memory representation of that identity. Given that familiar faces are represented in a more viewpoint-invariant manner as compared to unfamiliar faces (Armann et al., 2016), it would be meaningful to test if the robustness of face mask effect persists in a famous face recognition task.

When perceiving faces, not only do we try to decode identity, but also to read emotions. Emotion recognition is crucial from an evolutionary perspective because it helps us gauge threats and can also facilitate positive social interactions (Elfenbein et al., 2007). This biologically innate ability plays a crucial role in many facets of life. By dividing a face into a visible top half and invisible bottom half, the widespread use of surgical masks has hindered nonverbal communication (e.g. McCrackin et al., 2022; Mheidly et al., 2020), in light of the six universally recognised facial expressions of emotion: surprise, fear, disgust, anger, happiness, and sadness (Ekman & Friesen, 1971). Past studies have attempted to identify the specific diagnostic features that are most crucial to decode emotions by using feature extraction algorithms (Kotsia et al., 2008) or by partially occluding target faces either with the ‘bubbles’ technique (Gosselin & Schyns, 2001), niqab (Fischer et al., 2012; Leach et al., 2016). However, studies that employed these paradigms have produced mixed findings. Mouth occlusion has been shown to cause a greater decrease in facial expression recognition than occlusion of the eyes, rendering the interpretation of anger, fear, happiness, and sadness much more difficult to detect than expressions more solely dependent on the eyes such as disgust. While there is a high consensus that covering the lower face parts leads to reduced performance in decoding happy emotions, incoherent results have been reported for other emotional expressions. For example, (Bombari et al., 2013) found a high relevance of the eyes in detecting fear, whereas Kotsia et al. (2008) observed a high reliance on the mouth region.

To the best of our knowledge, only two recent studies directly measured the effect of face masks on emotion recognition ability. Carbon (2020) reported that when faces were partly covered by a face mask, people tend to misinterpret disgusted faces as being angry and misjudge other emotions such as happy, sad, and angry as neutral. Saunders et al. (2021) reported that masks change the transmission of sound and remove visible speechreading cues, as well as decrease the visibility of facial expressions. The authors acknowledged that masking limits our ability to perceive facial expressions expressing happiness as well as disgust, but recognized that it has less impact on our ability to recognize surprise, anger, and fear. This has been further illustrated in studies of women wearing Islamic face coverings such as a niqab or hijab (Fischer et al., 2012), as well as through studies systematically covering different areas of the face (Wegrzyn et al., 2017).

Gender classification—an ability to recognise a person based on the characteristics that differentiate between masculinity and femininity—has been the subject of extensive research due to its applications in applied research areas, such as the security and surveillance industry, mobile application, and human–computer interaction (Lin et al., 2016). This binary classification problem (male or female) is a relatively easy task for humans (Wild et al., 2000) but a challenging task for machines (Guo, 2012). Thus, gender classification based on facial images has received increased attention in the computer vision community but less in human research. Not only humans are often able to readily determine gender through visual inspection, but also the process can sometimes take place in the near absence of attention (Reddy et al., 2004). In fact, on the most basic characteristic, gender is a relatively invariant aspect of faces (Kaul et al., 2010). To categorise a face as male or female, one must be able to extract and encode facial information that it shares with all or most faces of the same gender. Humans tend to classify males or females based on the presence or absence of a moustache, beard and eyelids (Chen, 2011). However, human perception of gender can encounter difficulties when subjects wear scarfs or glasses, leading to the loss of information from important facial features. These findings offer a hint at a big challenge for humans to recognise gender information from face images if the lower half of faces is covered by a surgical mask. Given the current prevalence of face masks in everyday life, this necessitates testing people’s ability to classify gender using only the top halves.

Human age is another important personal attribute or trait, which can be directly inferred from facial appearance. Age estimation is the ability to label one’s face with an exact age or age group. Despite multiple research attempts at identifying cognitive and facial ageing patterns for accurate age estimation (Dehon & Brédart, 2001; Moyse, 2014; Short et al., 2019; Voelkle et al., 2012), automatic age estimation accuracies are still far below human accuracy (Angulu et al., 2018; Guo, 2012; Nguyen et al., 2014), mainly because facial ageing is caused by a wide variety of factors (e.g. skin texture, diet, and ethnicity) (Porcheron et al., 2017). In psychological research, age estimation has been studied to find out how people learn age-introduced patterns or variations (e.g. facial shape and texture) from the faces for accurate age estimation. The motivation to study age estimation arises from many aspects. Like facial emotional expressions, perceived age is another essential attribute derived from human facial appearance that can impact interpersonal behaviour in social settings. Also, the ability to determine age information from human faces has real-world applications, ranging from security control to forensic investigations. To date, there has been relatively little work on human age estimation, not to mention the impact of face masks on the ability to perceive the age of a person from the face. Thorley et al. (2022) recently found that, when the face mask was present, age estimates of unfamiliar faces increased by a median of 1.30 years. More specifically, Davis and Attard-Johnson (2022) reported that face masks lead to an overestimation of young females’ age, albeit huge individual differences in age estimation ability ranging from 1 to 20 years of the target’s actual age, suggesting a modulatory effect of gender.

### The current study

Although the underlying mechanisms of facial identification, emotion recognition, gender classification, and age estimation judgements have been investigated in the previous work, there is little empirical research on the effect of face masks on facial identification and social judgements per se. Extending previous work, the overarching aim of the current study was to systematically investigate the effect of surgical face masks on human performance in emotion recognition, famous face recognition, gender classification, and age estimation. By comparing performance levels of masked and unmasked faces, we evaluate the extent to which the facial information that remains in the upper halves contributes to these judgments. To address these questions, all participants completed four face tasks: famous face recognition, emotion recognition, gender classification, and age estimation tasks.

Numerous studies have reported evidence of females’ advantage over males in face processing (Herlitz & Rehnman, 2008). For example, McBain and colleagues (2009) found that the female advantage for face detection and face identity discrimination persists even under challenging conditions such as inverted faces and visual noise. Also, there is a well-established literature on the own-gender bias in face recognition (Heisz et al., 2013; Wright & Sladden, 2003) and emotion perception (Fischer et al., 2018). In this vein, several studies underline the advantage of women in decoding emotions, not only in static stimuli (Olderbak et al., 2018; Thompson & Voyer, 2014), but also in dynamic stimuli (Wingenbach et al., 2018). Despite the increasing number of studies testing the face mask effect on face processing, the gender differences in this correspondence have not been directly addressed. To fill this literature gap, in the present study, we also studied and analysed the potential gender effect for each face task.

### Hypotheses

For the *famous face recognition task*, despite the increased familiarity with the target faces, we predicted that participants would be less accurate in identifying celebrities’ faces when parts of the faces are covered. For the *emotion recognition task*, not only did we expect that participants would be less likely to accurately categorise emotion expression on a masked face as compared to an unmasked face, but also that the recognition accuracy of different emotions may depend on contrasting parts of the face: some emotions are better recognized from the bottom half while the others are better recognized from the upper half (Wells et al., 2016). More specifically, as people tend to confuse anger with disgust, the presence of a face mask might further deteriorate the recognition of anger and disgust. Happy and surprise—the two easiest expressions to recognise from the eye region—would be less affected by the presence of the face mask. Variation in the eye region is also crucial for distinguishing between fear (Adolphs et al., 2005) and surprise (Smith & Rossit, 2018), and therefore, these two emotions would be less affected by the presence of face masks.

For the *age estimation task*, one possibility is that wearing mask distorts configural information, which could influence age estimates, just as it influences emotion recognition (Carbon, 2020). If the bottom face halves contain more sources of information that observers use to extract age information as compared to the top halves, then the difference between perceived age and actual age would be larger when the target face is masked (vs. unmasked), regardless of the chronological age of the target stimuli. Also, we anticipated that *gender categorization*, an automatic, early processing stage, is less influenced by the occlusion of face mask as compared to other face tasks.

Although the face mask effects have been recently studied with different experimental paradigms, it remains largely unknown whether they are equivalent across gender. If females tend to focus more on the eyes (particularly in the left eyes) compared to males when processing faces (Coutrot & Guyader, 2014), then we would observe a less pronounced face mask effect in females than males. However, if females attend more to the internal features of the faces compared to males possibly due to a higher reliance on second-order properties of the face (Rennels & Cummings, 2013), but see (Knudsen et al., 2021; Mishra et al., 2019), their face processing would be more impaired by face masks.

## Methods

### Participants

A total of 110 participants were recruited online via Testable Minds (https://www.testable.org/; for details, see Rezlescu et al., 2020). Prior to the experiment, informed consent was obtained from each participant. This sample size was chosen to ensure a robust statistical power. A priori power analysis using G*Power 3.1.9.2 (Faul et al., 2007) indicated that, for all of the terms in our analyses that directly related to our hypotheses (all of which are within-subjects interactions in ANOVAs), an estimated sample size of 91 would give sufficient power to detect effect sizes of $\eta_{p}^{2}$ < 0.01 (a small effect size), with *α* = 0.05, and power (1 − *β*) = 0.80. While the median time and range of time taken to complete the experiment were 19.66 min and 14.39–22.97 min, respectively, two participants who took an unconscionably short duration (< 10 min) to complete the entire study, likely due to inattentiveness, were excluded from the analysis. The final sample consisted of 108 participants: 56 males (*M*_age_ = 32.39 years, SD = 11.36) and 52 females (*M*_age_ = 29.06 years, SD = 8.56). All experimental procedures and protocols were approved by the Science & Engineering Research Ethics Committee (SEREC) at the University of Nottingham Malaysia. All participants gave their informed consent prior to the experiment and were compensated with 3USD for their participation.

### Apparatus, stimuli and procedure

We obtained the facial photographs for the age estimation and gender classification tasks from the CAL/PAL face database (Minear & Park, 2004) and for famous celebrity and emotional photographs from the VISGRAF face database (https://app.visgraf.impa.br/database/faces/; as cited in Mena-Chalco et al., 2009). The colour photographs were cut out along the chin line and the outer contour of the head, showing only the face without any distinctive cues from clothing. Different images of surgical face masks collected online were superimposed over the original face stimuli using Adobe Photoshop™ CC 2019 (Adobe Systems Incorporated, San Jose, CA, USA). All face images were 400 × 550 pixels in size and were presented on a white background. The study was conducted online and programmed in Testable (https://www.testable.com) whereby the size of stimuli was scaled with the screen resolution of the participants’ screens. At the start of the experiment, participants were asked to match the length of a line to the length of a bank card. For the stimuli presented within each face task, an equal male–female gender split (half male, half female) was made. In each task, trials were completed in a single block and participants were given a short break between the four tasks. The order of block presentations was counterbalanced across participants. The whole experiment took roughly 30 min to complete.

#### Famous face recognition task

**Fig. 1:**
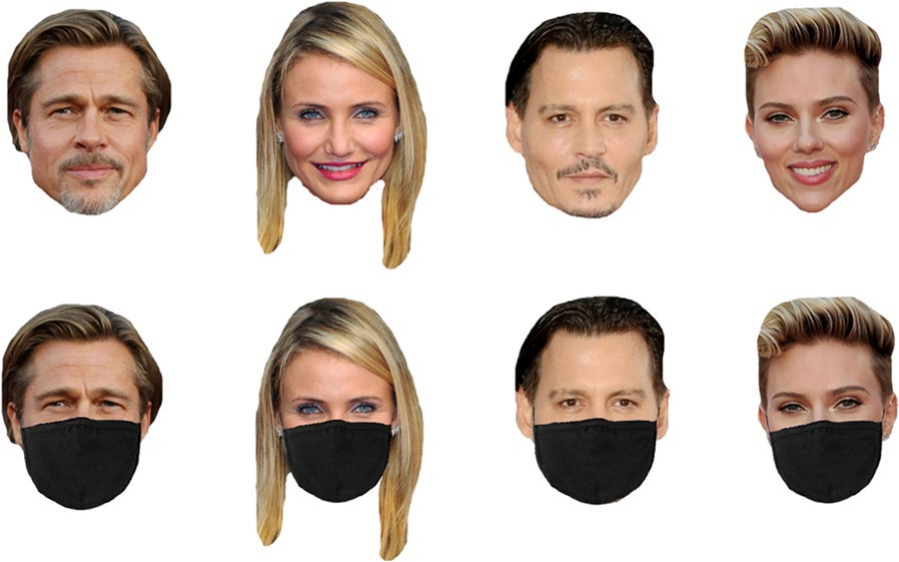
Example images used in the famous face identification task. From left to right: Brad Pitt, Cameron Diaz, Johnny Deep, Scarlett Johansson

Participants were presented with 20 famous faces and were asked to provide their names. Each celebrity's face was presented in two forms: masked or full face. Figure 1 shows the sample stimuli. If they knew the faces, but could not remember the name, they could provide other identity information (e.g. profession, movies, or songs associated with them). Each face was displayed for three seconds, then a box would appear at the bottom of the screen for participants to type in their answers. If they did not know the name or any identity information about the faces, they were required to write ‘I do not know this face’. They had to give their answers as quickly and as accurately as possible. Their level of recognition was assessed, along with their ability to retrieve the identity information. One point was given to each photograph correctly named. Incomplete name, distorted production or no response was scored zero. Each accurate identity information was scored 0.25, with a maximum score of 1.

#### Emotion categorisation task

**Fig. 2:**
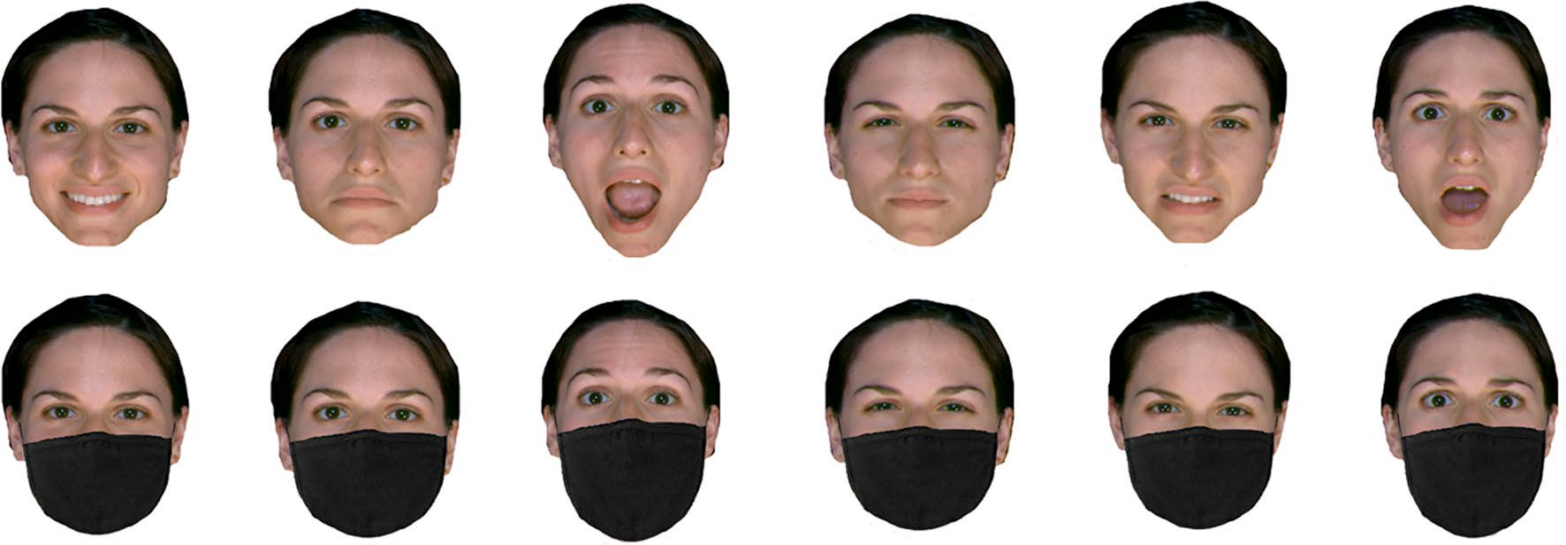
Sample images from the same identity showing six different emotions without a mask (top row) and wearing a mask (bottom row). From left to right: happy, sad, surprised, anger, disgust, fear. Original photographs were taken from VISGRAF face database

Since we used six different facial identities, and each identity portrayed six basic emotional expressions of full intensity, either with or without a mask, a total of 72 trials were presented. Each face stimulus displaying one of the six emotional expressions (see Fig. 2) was presented until a response was made. Participants were required to indicate the expressed emotion via a six-alternative forced-choice procedure by pressing the corresponding button (z = Happy, x = Sadness, c = Surprise, b = Anger, n = Disgust, m = Fear) on the keyboard. Each response was followed by an interstimulus interval (blank screen) of one second, which preceded the next face stimulus. The participants’ accuracy in recognising the emotions was measured.

#### Gender classification task

**Fig. 3:**
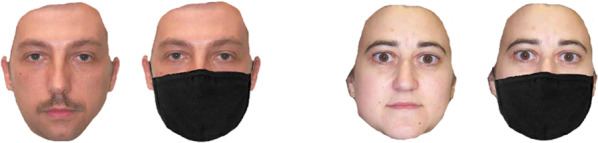
Gender stimuli of neutral expression with or without mask were used in the gender classification task

To avoid the ceiling effect caused by strong gender cues such as the presence of distinctive hairstyles, participants were presented with a series of faces without hair (see Fig. 3). All faces displayed a full-frontal neutral expression. Each trial started with a central fixation cross (presented for one second), followed by a face stimulus displayed until the participant rendered a judgment. The task was to classify 64 faces based on gender by pressing ‘F’ for female and ‘M’ for male, as quickly and as accurately as possible. The order of trial presentation was randomised across participants. Both accuracy and response time were recorded automatically.

#### Age estimation task

**Fig. 4:**
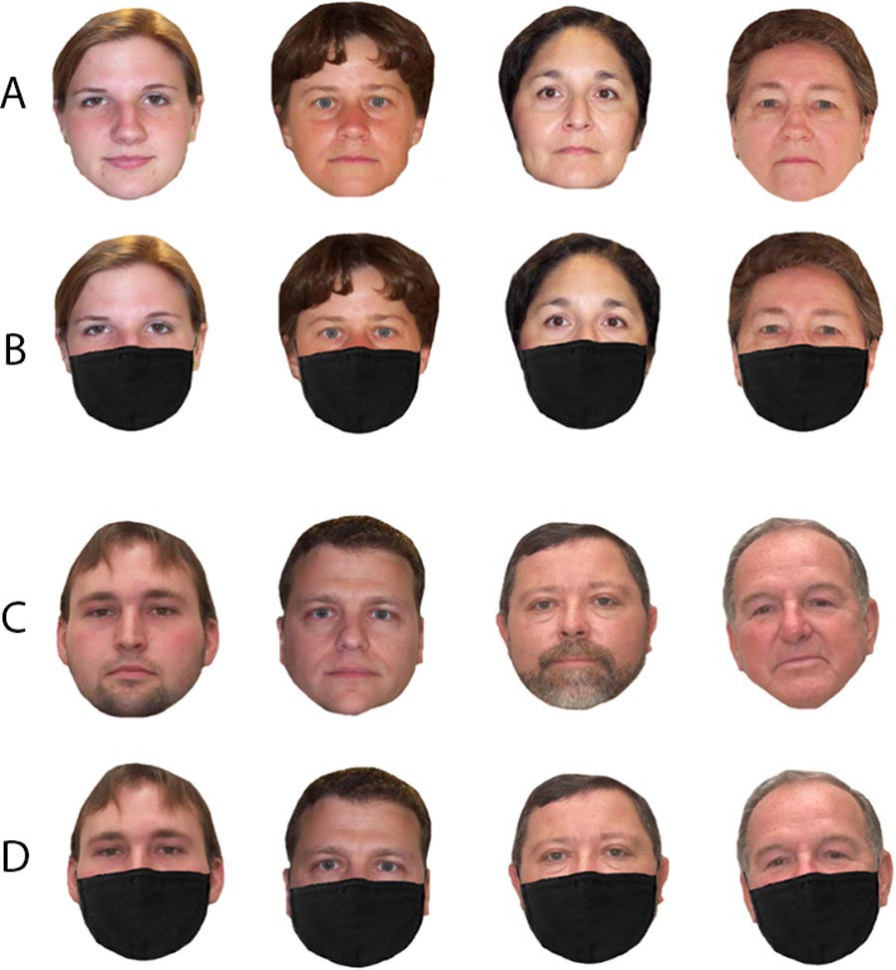
Samples from the VISGRAF database used in the age estimation task. Top panel shows unmasked (**A**) and masked (**B**) female faces; bottom panel shows unmasked (**C**) and masked (**D**) male faces. Age range ascends from left to right: 20–29, 30–39, 40–49 and 50–79

Each participant completed 64 trials in total, equal between male and female, at four age ranges: 20–29, 30–39, 40–49 and 50–79 (see Fig. 4). Half of the faces were presented with face masks, and each identity was presented twice. Participants were asked to rate the perceived age of the face using an integer entered on the number pad of a computer keyboard. They were not informed about the age range of the stimuli, and no feedback was given on the accuracy of their responses. On each trial, a single frontal view image was presented until the participant had typed their response in whole numbers. To prevent age estimates from being systematically biased towards the age of preceding face, the order of presentation was randomised across participants.

## Results

Considering that gender differences have been reported in face recognition and social judgements from faces (Cellerino et al., 2004; Heisz et al., 2013; Knudsen et al., 2021; Rennels & Cummings, 2013; Sartucci et al., 2004; Thompson & Voyer, 2014), we also included gender of participants as one of the variables. Both accuracies and mean reaction times for correct responses were recorded for each task. Statistical analyses were conducted using IBM SPSS version 26. Bonferroni-corrected *p*-values were reported for post hoc multiple comparisons.

### Famous face identification

A 2 (face type: masked or full faces) × 2 (participant gender: male versus female) × 2 (face gender: male versus female) ANOVA performed on the mean accuracy scores revealed a significant main effect of face type, *F*(1, 106) = 13.59, *p* < 0.001, $\eta_{p}^{2}$ = 0.11, whereby familiar faces without face masks (*M* = 0.65, SD = 0.31) were identified better than those wearing face mask (*M* = 0.61, SD = 0.31). There was a significant interaction between face type and participant gender, *F*(1,106) = 3.91, *p* = 0.017, $\eta_{p}^{2}$ = 0.04. Male participants identified unmasked faces (*M* = 0.64, SD = 0.25) better than masked faces (*M* = 0.57, SD = 0.25) (*p* < 0.001). In contrast, female participants performed equally well for unmasked faces (*M* = 0.67, SD = 0.28) and masked faces (*M* = 0.65, SD = 0.28). The main effect of face gender was significant, *F*(1,106) = 60.66, *p* < 0.001, $\eta_{p}^{2}$ = 0.36, such that male faces (*M* = 0.70, SD = 0.31) were identified more accurately than female faces (*M* = 0.56, SD = 0.31).

**Fig. 5:**
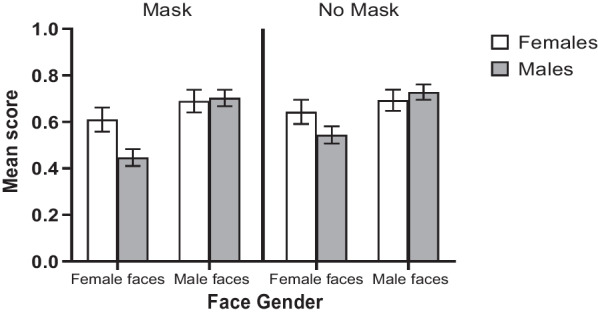
Average accuracy scores of male and female observers for masked and unmasked faces by face gender in the famous face identification task. Note: *p* > 0.05(ns), *p ≤ 0.05 (*), **p ≤ 0.01(**), *p* ≤ 0.001(***)

A significant interaction between face gender and participant gender was found (see Fig. 5), F(1, 106) = 18.14, *p* < 0.001, $\eta_{p}^{2}$ = 0.15. Female participants identified female faces (*M* = 0.63, SD = 0.28) better than male participants (*M* = 0.50, SD = 0.31) (*p* = 0.03), whereas male participants (*M* = 0.72, SD = 0.28) did not perform significantly better than female participants (*M* = 0.69, SD = 0.28) for male faces (*p* = 0.66). More interestingly, the interaction between face type and face gender reached significance, *F*(1, 106) = 5.84, *p* = 0.02, $\eta_{p}^{2}$ = 0.05, revealing that female faces were harder to identify when they were masked (*M* = 0.53, SD = 0.33) than when presented in whole (*M* = 0.59, SD = 0.33) (*p* < 0.001). In contrast, no difference between masked (*M* = 0.70, SD = 0.31) and control condition (*M* = 0.71, SD = 0.29) (*p* = 0.30) was found for male faces. Neither the main effect of participant gender, *F*(1,106) = 0.90, *p* = 0.35, nor the three-way interaction was significant, *F*(1, 106) = 1.07, *p* = 0.30.

### Emotion recognition

**Fig. 6:**
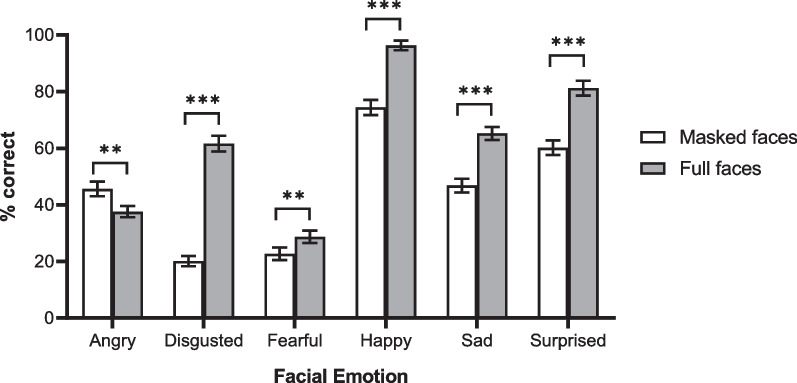
Mean percentage scores for all emotion expressions for masked and full faces. Error bars represent standard error of the mean. *Note:* For the ease of illustration, the scores are presented in percentages instead of proportions. In this six-alternative force choice task, the chance level performance for is 16.67% instead of 50%. ***p < 0.001, **p ≤ 0.01

Overall performance for correctly identifying facial emotions in faces with masks was remarkable (mean accuracy = 44.97%) with no participant performing below the chance level of 16.67%. A mixed ANOVA was performed on the recognition accuracy (i.e. percent correct scores) with both face type and emotion expression as within-subjects variables, and the gender of participant as a between-subject variable. The analysis revealed a main effect of face type, *F*(1, 106) = 273.01, *p* < 0.001, $\eta_{p}^{2}$ = 0.72, such that emotional expressions were better identified from full faces (*M* = 61.77%, SD = 14.24%) than masked faces (*M* = 44.97%, SD = 12.26%). There was a significant main effect of gender of participant, *F*(1, 106) = 3.00, *p* = 0.04, $\eta_{p}^{2}$ = 0.03, signalling that females (*M* = 56.67%, SD = 14.82%) scored higher than males (*M* = 52.90%, SD = 10.55%). The main effect of facial emotion was significant, *F*(5, 530) = 151.32, *p* < 0.001, $\eta_{p}^{2}$ = 0.59. Participants were best at recognising Happy (*M* = 85.36%, SD = 20.06%), followed by Surprise (*M* = 70.72%, SD = 24.21%), Sad (*M* = 55.96%, SD = 21.20%), Anger (*M* = 41.61%, SD = 20.26%), Disgust (*M* = 40.86%, SD = 19.23%), and Fear (*M* = 25.69%, SD = 19.75%) (*p* < 0.001 for all pairwise comparisons, except for the difference between anger and disgust where *p* = 1). More interestingly, a significant interaction between facial emotion and face type was found, *F*(5, 530) = 45.82, *p* < 0.001, $\eta_{p}^{2}$ = 0.30. While disgusted (*p* < 0.001), fearful (*p* = 0.01), happy (*p* < 0.001), sad (*p* < 0.001), and surprised (*p* < 0.001) faces were being identified more accurately in full faces than in masked faces, angry faces (*p* = 0.002) showed the opposite pattern (see Fig. 6). That is, angry expressions were better identified in masked faces than in full faces. However, no significant three-way interaction was found, *F*(5, 530) = 0.47, *p* = 0.80.

### Gender Classification

**Fig. 7:**
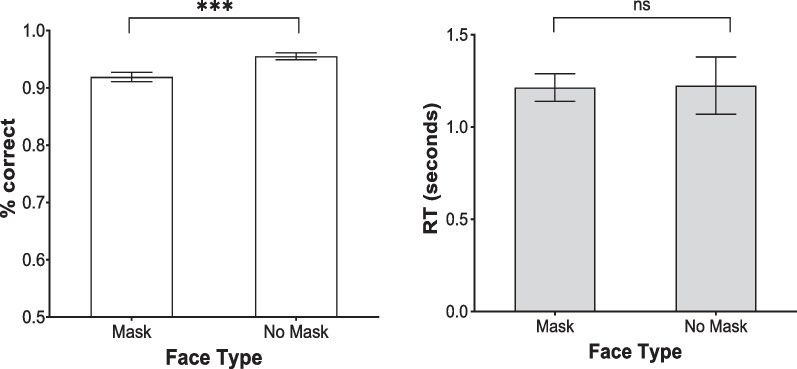
The percentage correct and mean RT for masked and unmasked faces in the gender classification task

A 2 (face type) × 2 (face gender) × (gender of participant) ANOVA showed a significant main effect of face type, *F*(1, 106) = 19.43, *p* < 0.001, $\eta_{p}^{2}$ = 0.16, such that full faces (*M* = 95.50%, SD = 6.24) were classified better than masked faces (*M* = 91.90%, SD = 8.31). The main effect of face gender was also significant, *F*(1, 106) = 84.29, *p* < 0.001, $\eta_{p}^{2}$ = 0.44, such that male faces (*M* = 98.91%, SD = 3.12) were classified better than female faces (*M* = 88.50%, SD = 11.43). In addition, there was a significant interaction between face type and face gender, *F*(1, 106) = 21.11, *p* < 0.001, $\eta_{p}^{2}$ = 0.17. Female faces were classified better when unmasked (*M* = 92.10%, SD = 15.59%) than masked (*M* = 84.90%, SD = 12.47%), but no difference between unmasked (*M* = 98.90%, SD = 4.37%) and masked faces (*M* = 99%, SD = 3.64%) was found for male faces. No significant main effect of participant gender was detected, *F*(1, 106) = 0.91, *p* = 0.34. The interaction between face type and gender of participant reached significance, *F*(1, 106) = 5.10, *p* = 0.03, $\eta_{p}^{2}$ = 0.05, accompanied by a significant three-way interaction, *F*(1, 106) = 3.88, *p* = 0.05, $\eta_{p}^{2}$ = 0.04. Split analyses showed that the participant gender × face type interaction was significant for female faces, *F*(1, 106) = 5.01, *p* = 0.03, $\eta_{p}^{2}$ = 0.05, but not for male faces, *F*(1,106) = 0.25, *p* = 0.62. Based on simple main effect analysis, male participants performed worse at classifying female faces presented in masked condition (*M* = 82.29%, SD = 14.42%) than when in full face type (*M* = 92.88%, SD = 11.88%) (*p* < 0.001). In contrast, female participants performed equally well at classifying female faces, regardless of whether they were masked (*M* = 87.50%, SD = 14.40%) or unmasked (*M* = 91.32%, SD = 11.85%) (*p* = 0.13) (Fig. 7).

### Age estimation

**Fig. 8:**
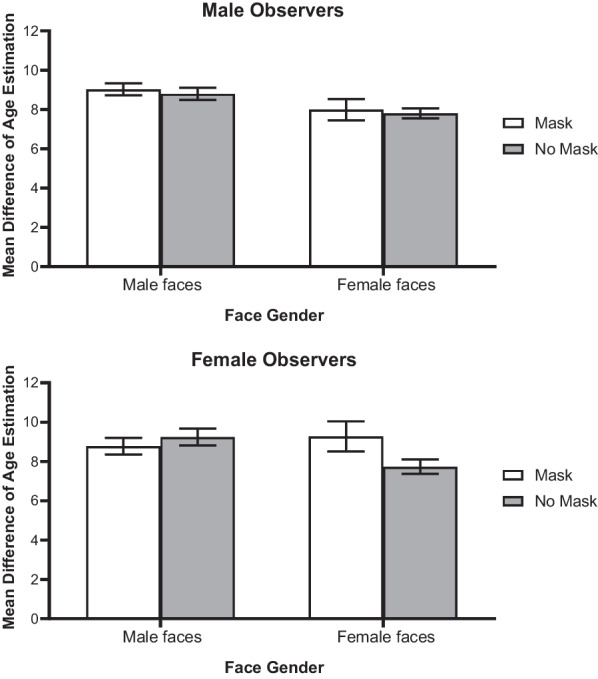
The mean difference of age estimation between male and female observers on own- versus other-gender faces presented with or without masks

For each photograph, the actual age of the face was subtracted from the participant’s estimation. We obtained the difference between the estimation and the actual age of the person presented on the photograph. This deviation was considered as an absolute value. We characterised how much the estimation deviated from the actual age of a face, regardless of whether it was an overestimation or underestimation of the age. These estimation errors were averaged for each face type (masked vs. full face). A 2 × 2 × 2 mixed factorial ANOVA was performed on these scores taking the gender of participant as between-subjects variable, and the face type and the face gender as within-subjects factors. Neither the main effect of face type, *F* (1, 105) = 2.45, *p* = 0.12, nor the main effect of gender of participant, *F*(1, 105) = 0.62, *p* = 0.43, was significant. However, there was a significant main effect of face gender, *F*(1, 105) = 6.15, *p* = 0.02, $\eta_{p}^{2}$ = 0.06, in which female faces (*M* = 8.20, *SE* = 0.28) were better estimated than the ages of male faces (*M* = 8.96, *SE* = 0.25) (*p* = 0.009). Additionally, a significant interaction between face type and face gender was found, *F*(1, 105) = 4.29, *p* = 0.04, $\eta_{p}^{2}$ = 0.04, qualified by a significant three-way interaction between face type, face gender, and gender of participant, *F*(1, 105) = 4.64, *p* = 0.03, $\eta_{p}^{2}$ = 0.04. Split analysis by face gender showed that the interaction between face type and participant gender was significant for male faces, *F*(1, 105) = 4.34, *p* = 0.04, $\eta_{p}^{2}$ = 0.04, but not for female faces, *F*(1,105) = 2.00, *p* = 0.16 (Fig. 8). Pairwise comparisons revealed that, regardless of the gender of participants, the ages of female faces were less accurately estimated when the bottom half was occluded (*M* = 8.42, SD = 4.61) than when full faces were presented (*M* = 7.78, SD = 2.17) (*p* = 0.05). In contrast, when estimating the ages of male faces, male participants showed the tendency to estimate worse when they were masked (*M* = 8.88, SD = 3.65) (*p* = 0.08) than when the faces were unmasked (*M* = 8.39, SD = 3.48), whereas females participants performed equally well for masked (*M* = 9.02, SD = 1.86) and unmasked male faces (*M* = 8.79, SD = 1.86) (*p* = 0.24).

## Discussion

While wearing a face mask has become common during the COVID-19 pandemic, the current study investigated whether, and if so, to what extent face masks interfere with famous face identification, emotion recognition, gender classification, and age estimation. Although these processes tap on different components of Bruce and Young’s (1986) model; however, if the presence of face mask generally impairs performance in all these tasks, such a robust face mask effect would imply that the occlusion of bottom face halves disrupts a common process—e.g. the structural encoding—where various primary facial attributes are encoded in early visual processing stages.

In the famous face identification task, our results clearly show that wearing a mask significantly impairs human performance even when recognising well-known celebrity faces. Notably, the magnitude of the face mask effect we found was much larger than that of Noyes et al. (2021) who employed an unfamiliar face matching task, as well as Carragher and Hancock (2020) who used a familiar face matching task. As matching faces is a relatively easy task as compared to the current famous face recognition-and-naming task, the actual face mask effect could have been understated the potential effect of face mask on face identifications. Consistent with our predictions, the presence of face masks resulted in a significant impairment (4% lower on average) to famous face identification. This result is not surprising, given that the mouth and nose are less useful for identification than the features of the upper face (Davies et al., 1977) and familiar face recognition is typically robust to various types of disruptions that impair performance for unfamiliar faces (Noyes & Jenkins, 2019). Such an observation was consistent with Carragher and Hancock’s (2020) recent findings from a face matching study which demonstrated that the presence of the face masks interrupted human performance in recognising both familiar and unfamiliar faces. Even with relatively high quality of facial representations in memory, participants generally had difficulties using only information from around the eye regions to discriminate and recognise familiar faces.

The reduction in performance could be attributable to the disruption of holistic face processing (Maurer et al., 2002; Tanaka & Farah, 1993; Wong et al., 2021)—whereby individual features (eyes, nose, mouth) are integrated into a coherent unified whole—as the spatial relationships between the core facial features were disrupted by the presence of face mask (Freud et al., 2020). Another source of evidence for the role of the lower part of the face in holistic face recognition comes from the difficulty recognising the identity of one of the face halves when they are aligned than when they are misaligned (Hole, 1994; Taubert & Alais, 2011), which is known as the *composite face effect* (Young et al., 1985). When the two face halves are misaligned, the halves cannot be perceived as a whole face and therefore a featural processing must be employed. Converging findings from neurophysiological (e.g. Jacques & Rossion, 2009, 2010) and neuropsychological studies (Avidan et al., 2011; Liu & Behrmann, 2014; Palermo et al., 2011) support that the composite face effect interferes with the structural encoding of faces. In a similar vein, the occlusion of the lower part of the face has been found to disrupt of holistic processing, which is essential for extracting facial cues for identity (Freud et al., 2020), emotion (Chen & Cheung, 2017; but see Murray et al., 2018), gender (Zhao & Hayward, 2010; Chen et al., 2018), and age (Gray et al., 2020; Hole & George, 2011).

In the current study, we also sought to examine participants’ ability to judge facial emotion using cues from the top face halves (i.e. the eye region) alone. Our findings resonate well with previous reports that anger, surprise, and sadness (Guarnera et al., 2015) can be readily recognised from the eye area than from the mouth area, but are inconsistent with a recent report that all emotional expressions were harder to read in faces with masks, except for fearful and neutral faces (Carbon, 2020). As expected, our results demonstrated that relative to full faces, emotional expressions on masked faces were less identifiable. While our analyses suggest a clear impaired performance for happy, surprised, sad, disgusted and fearful expressions, the opposite direction was found when observers were required to identify angry faces. Participants were generally better at recognising anger from masked faces than that from full faces, indicating that not only the eyes region may provide most of the emotional information indicative of this threat-relevant emotional state, but also that the mouth region might lead to confusion between expressions of anger and disgust (Wegrzyn et al., 2017). This type of evidence is consistent with the functional-evolutionary notion that the human visual system is particularly sensitive to threatening angry expressions to ensure survival (Fox et al., 2000). More interestingly, although the presence of face masks hinders actual information transmission, it strengthens our perceptions of angry emotions produced by frowning and such an increase in sensitivity could be elicited by negative feelings of unfriendliness and emotional distancing (Grundmann et al., 2021).

Such a novel finding has an important implication. Recognition of and response to the outward emotional displays of one's peers is a critical and necessary component of social interaction as it helps individuals to modify their behaviour to align with social communication and norms. When these emotional displays are inhibited by physical barriers such as masks, our ability to communicate effectively with one another is drastically limited and we are primarily left with mimicking negative (frown) emotions. With the lifting of COVID-19-inspired restrictions, the negative psychosocial effects of masks on facial emotion perception should be addressed carefully to extenuate social isolation resulting from facial feature occlusion.

Here, we also investigated whether face masks would impair the performance of judging one’s gender and age. Results revealed a discernible effect of mask-wearing on the accuracy (≈ 5% reduction on average) with which gender decisions were made, indicating that the top halves provide stronger gender cues to make this decision with relative ease, as compared to the bottom halves. While there is sufficient structural information in the top halves for accurately determining face gender, the performance level was modulated by the face gender: female faces were classified better when unmasked than masked while such a difference was not found for male faces. This seems to suggest the nose and mouth regions (e.g. chin, cheek, lips) on female faces may provide more complementary and discriminative information (e.g. facial femininity) in addition to the eye region compared to male faces.

In line with Thorley et al. (2022) and Davis and Attard-Johnson (2022), the current study provides evidence that the presence of a face mask harms age estimation ability, but to a small extent. As compared with full faces, partial face occlusion by mask tends to produce more inaccurate age estimates. More interestingly, for male faces, female participants recognised masked and unmasked equally well; for female faces, regardless of participant gender, were evaluated more poorly when they were masked than unmasked, suggesting that the bottom face halves could be more informative for estimating age of a female than a male. We did not expect these findings, but when considering evidence that the jaw, chin, and zygoma (cheekbone) are collectively crucial for the perception of facial femininity (Li et al., 2021; Mogilski & Welling, 2018), it is plausible the presence of face mask restricts the use of these perceptual cues for age estimation. This raises the question of the relative quality of age information used by eyewitnesses in their descriptions when a face was partially covered by a mask. Caution should be reinforced when treating eyewitnesses' statements regarding, at least, the age of another person since in this case, they are more likely to be inaccurate than if they were determining the age of a person who does not wear a face mask.

Concerning gender differences, there was an evident female advantage over males in the famous face identification and emotional recognition tasks (see Lewin & Herlitz, 2002). In contrast to males, female participants were able to recognise familiar faces equally well, with or without a mask. The female advantage was even evident when conscious processing of the face identities was restricted by the presence of face mask. Such a female advantage could be attributed to a more effective featural processing, as indicated by better performance with inverted faces in females compared to males (McBain et al., 2009). Besides, females seem to have a greater ability to perceive facial emotions at an automatic processing level compared to males (Abbruzzese et al., 2019; Saylik et al., 2018; Wingenbach et al., 2018), even when asked to categorise emotion using cues from the top face halves only. Recent research (Olderbak et al., 2018) conducted on a large community sample of persons ranging from younger than 15 to older than 60 years of age highlighted a higher ability in females of all ages to read emotional faces. High perceptual sensitivity to minimal social-affective signals may contribute to female advantage in understanding other persons' emotional states (Donges et al., 2012). The greater ability of females to correctly recognise familiar faces and emotions could be tied to their scanning behaviour at encoding (Heisz et al., 2013; Rennels & Cummings, 2013) and their normative role as the primary caregiver for their children and within families (Babchuk et al., 1985). Being able to correctly and rapidly identify the emotion expressed by others would facilitate the understanding of emotional states during social interactions. This facilitation would allow for responding to the needs of the others rapidly and appropriately. For example, recognising sadness in another person can lead to comforting behaviour, which is important for bonding and nurturing roles. Fast and automatic processing of facial emotion by females might involve innate mechanisms designed through evolution to facilitate effective caring of offspring, as proposed by the *primary caretaker hypothesis* (Babchuk et al., 1985).

In the gender categorisation and age estimation task, we observed a significant own-gender advantage for female but not for male participants. Regardless of face type, female participants were generally better at categorising own-gender faces, as compared to male participants. These findings are consistent with the previous report that females have an advantage in face processing (Herlitz & Lovén, 2013), sometimes for female over male faces (Cellerino et al., 2004; Rehnman, 2007). Female own-gender bias in gender categorisation and age estimation may stem from early perceptual expertise for female faces, which may be strengthened by reciprocal interactions and psychological processes directing girls’ and women’s interest in other females (Herlitz & Lovén, 2013; Lovén et al., 2011). Further research could attempt to replicate our results—both female advantage and own-gender bias—to confirm the potential modulatory influence of gender on the face mask effect.

The present research raised concerns about the negative effects of wearing face masks in social settings. COVID-19 brought to the world more than just widespread disease but also a radical change in human communication. By cutting the visual surface areas of our faces in half, masks make it incredibly challenging to perceive each other’s facial expressions which are critical and necessary components of social interaction. This may cause individuals to rebel much more over being forced to wear face masks. The impact of face masks on social judgments and face identification may be particularly worrisome in settings where accurate emotion recognition and establishing relationships are pivotal (e.g. health and education sectors). While our results highlight the side effects of face masks on social functioning, these findings should not be put forward as an argument for advocating the freedom of choice of wearing face masks.

The experiment presented here contained samples of Caucasian participants only, using Caucasian faces, which limits the generalisability of our results to other cultures. Recent studies have hinted at cultural differences in the embracement of wearing masks, and some cultures read emotion differently compared to those in the West (Cossio et al., 2012; Fang et al., 2019; Gul & Humphreys, 2014; Prado et al., 2014). Yet, to the best of our knowledge, no studies have directly tested the face mask effects using a cross-cultural design. Further studies involving non-Western populations are therefore required to assess the robustness of the face mask effects reported here. Also, an important methodological caveat should be noted before drawing conclusions from the current findings. The use of intermixed method in presenting masked and unmasked faces may increase participants’ reliance on a pictorial matching strategy based on the top face half, mitigating the magnitude of face mask effects. This concern is supported by recent studies which demonstrated that most observers are capable of matching masked faces with remarkably high sensitivity (Estudillo et al., 2021) and developing strategies for coping with the unwarranted effects of face masks in blocked- than in mixed designs (Fitousi et al., 2021).

In a society where face-to-face communication is inevitable, it becomes crucial to find solutions to our diminished ability to recognise faces and to communicate via facial expressions that are hidden under a mask barrier. One intriguing question that arises from our work involves the malleability of our face mechanisms to rapidly adapt to this era of face masks, employing strategies that identify faces just using the area around the eyes. If wearing masks distorts face processing due to the partial occlusion and/or the brain fills in the missing features, it would be interesting to know how the brain forms the representation when a protective mask occludes the bottom face halves. Yet, Freud et al. (2020) recently found no evidence of improvement in face perception abilities of masked faces following extensive, naturalistic exposure to occluded faces over time, suggesting the rigidity of matured face processing system.

## Conclusions

In summary, while face masks may effectively curb the spread of virus, the current study demonstrated that they have collateral consequences, undermining not only face identification but also social judgments on one’s emotion, age, and gender. It must be noted that these findings should not be taken as a reason for not wearing masks in situations where they are of medical use, particularly during the COVID-19 pandemic. More broadly, these findings serve to implicate the critical importance of societal adaptation to the advent of mask-wearing in ways that minimise the psychosocial impact it induces. Future research should focus on how we can encourage the better adoption of masks usage by developing coping strategies and skills that can ease our communication with face masks, which is crucial in our efforts to navigate the COVID-19 pandemic and any other pandemic that might erupt in the future (Scudellari, 2020; Walsh et al., 2020).

## Data Availability

The datasets used and/or analysed during the current study are available from the corresponding author on reasonable request.
